# Functionalized Siloxane Coating as Protection of the Surface of Cement Composites Against Phototropic Colonization

**DOI:** 10.3390/ijms27031586

**Published:** 2026-02-05

**Authors:** Joanna Karasiewicz, Marta Thomas, Paulina Nowicka-Krawczyk, Rafał M. Olszyński, Piotr K. Zakrzewski, Agnieszka Ślosarczyk

**Affiliations:** 1 Faculty of Chemistry, Adam Mickiewicz University in Poznan, Uniwersytetu Poznańskiego 8, 61-614 Poznan, Poland; 2 Faculty of Civil and Transport Engineering, Institute of Building Engineering, Poznan University of Technology, Piotrowo 5, 60-965 Poznan, Poland; marta.thomas@put.poznan.pl (M.T.); agnieszka.slosarczyk@put.poznan.pl (A.Ś.); 3 Department of Algology and Mycology, Faculty of Biology and Environmental Protection, University of Lodz, Banacha 12/16, 90-237 Lodz, Poland; paulina.nowicka@biol.uni.lodz.pl (P.N.-K.); rafal.olszynski@biol.uni.lodz.pl (R.M.O.); 4 Department of Molecular Neurochemistry, Faculty of Health Sciences, Medical University of Lodz, Mazowiecka 6/8, 92-215 Lodz, Poland; piotr.zakrzewski@umed.lodz.pl

**Keywords:** siloxane-based coating, protective coating for concrete, biofouling of concrete

## Abstract

This article presents the concept of using a functionalised siloxane compound HOL9 with amphiphilic properties as a coating for cement composites to enhance their antifouling properties against algae. The biological properties of the compound were assessed based on its ability to inhibit chlorophyll fluorescence intensity, which is used as an indicator of photosynthetic activity and biofilm development. The greatest decrease in algal photosynthetic activity was observed for a 10% aqueous solution of HOL9 applied by painting. In these conditions, the maximum ^chl^FI value decreased by 97.6%. In addition, the impact of the protective coating containing HOL9 on the fundamental physical and mechanical characteristics of the cement composite, along with its resilience to frost cycling, was thoroughly investigated. The coating applied by immersion demonstrated a 50.7% strength loss after 150 freeze–thaw cycles, while the coating applied by painting exhibited a 43.8% loss. In comparison, the control samples experienced a 42.8% strength reduction. It has been demonstrated that the method of application, the modifier concentration, and the type of solvent can have a substantial impact on the protective properties of concrete. The most marked inhibition of algae photosynthetic activity was observed with a 10% aqueous solution applied by painting.

## 1. Introduction

Cement-based composites boast a multitude of advantages, including elevated compressive strength, durability, and fire resistance. Their adaptability to diverse forms and integration into sustainable development practices, marked by the capacity for recycling and the combination of cement composites with various types of waste, further underscores their environmental benefits. The durability of the cement composite is contingent upon its resilience against deleterious external factors that precipitate various forms of corrosion. One form of corrosion that can manifest on the surface of the cement composite is biological corrosion. The phenomenon of biological corrosion, precipitated by the proliferation of algae, has been demonstrated to exert a deleterious effect on the durability of cement composites. This phenomenon is of particular concern within the context of marine and coastal environments. The process of Micro-biologically Influenced Deterioration (MID), initiated by the presence of algae, involves the extraction of essential components such as calcium, silica, and magnesium from the cement matrix. These elements are indispensable for the algae’s metabolic processes. This results in the formation of cracks, which are particularly dangerous for reinforced concrete, as they can also lead to the development of microbiologically influenced corrosion (MIC) of concrete reinforcement elements [1,2]. The presence of algae can create a habitat for other organisms that cause concrete deterioration and reinforcement steel corrosion. It has been demonstrated that such organisms may be either fungi or acid-producing, sulfur-oxidizing bacteria that form a biofilm on the surface of the material in conjunction with algae [3,4]. It has been documented that algae have the capacity to colonize concrete surfaces, adhere to them, and produce metabolites, including organic acids. These metabolites contribute to the biodeterioration of the concrete structure by leaching components of the cement matrix and weakening its mechanical properties [5,6]. Recent studies on concrete bio-receptivity have demonstrated that the microstructure and surface features of cementitious materials significantly influence the attachment and development of phototrophic biofilms, thereby enabling algae and fungi to establish more stable communities on the substrate [6,7]. Furthermore, the presence of phototrophic biofilms involving algae has been shown to increase the transport of moisture and nutrients into the concrete, thereby promoting further biodegradation processes and leading to deterioration of the surface properties of the material [7,8]. Cement matrix corrosion can also be caused by fungi, such as *Aspergillus flavus*, *Mucor*, and *Aureobasidium pululans*. These organisms can form biofilms and mycelia on the cement composite surface, which can lead to both physical and chemical damage [9,10]. The presence of biofilms on the surface of cement composites is a common occurrence. The presence of microorganisms on the surface of building materials can compromise the integrity of the cement matrix [11]. A plethora of methodologies exist for the surface cleaning of biofilms, including mechanical removal, water and surfactant solution washing, and the use of laser UV or IR irradiation for biofilm removal. To mitigate the adhesion and biofilm formation that are hallmarks of such infections, a range of hydrophobic agents and polymer-based compounds can be utilized as protective measures. A combined method involves the utilization of biocides, which have been shown to result in the destruction of algal cells, the inhibition of photosynthesis, and the prevention of biofilm formation [12]. While biocides are the most effective method of cleaning and protecting materials from the development of biocorrosion, their negative impact on both human health and the environment constitutes their most significant drawback [13,14,15]. Additionally, the utilization of polymer-based agents may give rise to concerns regarding their safety for humans and the environment [16]. Furthermore, certain studies have indicated that polymer-based agents exhibit effective protection against moisture, yet their efficacy in combating biodeterioration may not be consistently adequate [17]. Another problem that has been observed to affect both polymer-based and hydrophobizing agents is a decrease in performance over time [18]. It is also imperative to acknowledge the potential impact of hydrophobic treatment on material properties, including alterations in appearance and other physical characteristics [19]. A second group of agents that prevent biodeterioration are silanes and siloxanes [20,21,22,23,24]. The effectiveness of these agents is related to their low viscosity and ability to spontaneously polymerize, which allows them to penetrate the pores of the cement matrix [25]. Li et al. demonstrated that a siloxane-based coating can provide long-lasting hydrophobicity to concrete surfaces, thereby preventing the adhesion of marine microorganisms and effectively inhibiting the formation of microbial biofilms [24]. Furthermore, Boutamart et al. demonstrate the feasibility of applying a self-cleaning coating to concrete surfaces through the use of a siloxane coating, which exhibits resistance to prolonged UV radiation, abrasion, and humid and warm environments [23]. Silicone polyethers represent a prominent class of modified polysiloxanes, a distinction that is attributable to their structural characteristics [26,27]. The absence of a substituent on every second silicon atom renders the compound vapour and gas permeable, a property that is crucial for the protection of building and construction materials [28,29]. Furthermore, the presence of polyether groups, which are capable of binding with terminal hydroxyl groups attached to the siloxane, facilitates the permanent binding of the active ingredient to the substrate or cement composite [21]. The most significant, effective, and widely utilized approach for incorporating a functional group, such as a polyether, into a polysiloxane chain involves the addition of a Si-H bond to unsaturated compounds that contain the functional group [30]. This process is referred to as hydrosilylation [26,31,32]. The raw material containing the Si-H group may be a siloxane polymer, a cyclic siloxane oligomer, or a monomeric silane with an Si-H bond and two hydrolysable substituents on the silicon atom [33]. These reactions are most often catalyzed by platinum compounds. The synthesis of silicone polyethers via hydrothiolation is also frequently described in the literature [26].

A body of research on modified siloxane coatings suggests that functional siloxane compounds have the capacity to impede microalgal colonization and their photosynthetic activity while preserving the mechanical properties of concrete. This finding indicates their potential application as protective agents against biodeterioration. Recent research has demonstrated that a newly formulated siloxane admixture (L43) effectively suppresses algal photosynthetic cell activity on cement composites without compromising the material’s strength or resistance to freeze–thaw cycles. These findings underscore the efficacy of functional siloxanes in delivering targeted biological corrosion protection [34]. The cement surface was coated with solutions containing functionalized siloxane with polyether groups. Subsequently, a series of rigorous evaluations were conducted on the substrates, encompassing biological, surface, and strength assessments. In this publication, we seek to address the question of whether the proposed HOL9 polyether siloxane can effectively protect the substrate against phototrophic colonization and biofilm development on the cement surface without compromising critical performance parameters such as frost resistance or water resistance.

## 2. Results

### 2.1. Results of Synthesis

**Scheme 1:**
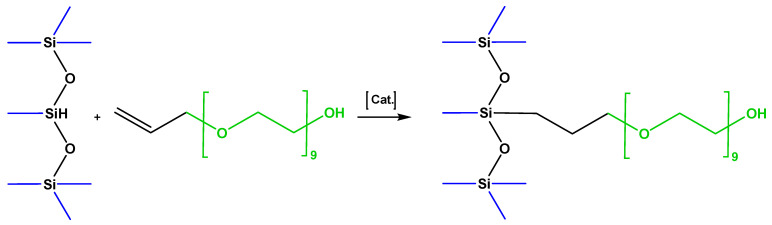
Hydrosilylation of allyl ether BIKANOL9 with 1,1,1,3,5,5,5-heptamethyltrisiloxane.

The siloxane polyether was synthesized using an allyl polyether containing nine ethoxy groups and a terminal hydroxy group. This process yielded a compound that exhibited amphiphilic characteristics. In addition to the hydrophobic methyl groups (blue lines in Scheme 1), hydrophilic poly-ether groups (green lines in Scheme 1) were attached to the silicon. It is imperative to note that all reactions were carried out using toluene as a solvent. The catalyst utilized in this study was the commercially available Karstedt catalyst. The hydrosilylation reaction is illustrated in Scheme 1.

The isolated product was subjected to spectroscopic analysis (^1^H NMR, ^13^C NMR, ^29^Si NMR) whose results are presented below:

**^1^H NMR** (CDCl_3_, TMS) δ (ppm): −0.13 (-Si(CH_3_); −0.02 (-Si(CH_3_)_3_); 0.33 (-SiCH_2_-); 1.44 (-CH_2_CH_2_CH_2_-); 3.24 (-CH_2_O-); 3.40–3.52 (-OCH_2_CH_2_-); 3.51 (-OH).

**^13^C NMR** (CDCl_3_, TMS) δ (ppm): −0.74 (-SiCH_3_); 1.49 (-Si(CH_3_)_3_); 13.11 (-SiCH_2_-); 22.79 (-CH_2_CH_2_CH_2_-); 61.19 (-CH_2_O-); 70.18 (-OCH_2_CH_2_-).

**^29^Si NMR** (CDCl_3_, TMS) δ (ppm): −21.76 (-Si(CH_3_)CH_2_); 6.98 (-Si(CH_3_)_3_).

The FT-IR spectrum of the HOL9 product is displayed in Figure S1, which is available as Supplementary Materials.

**Figure 1:**
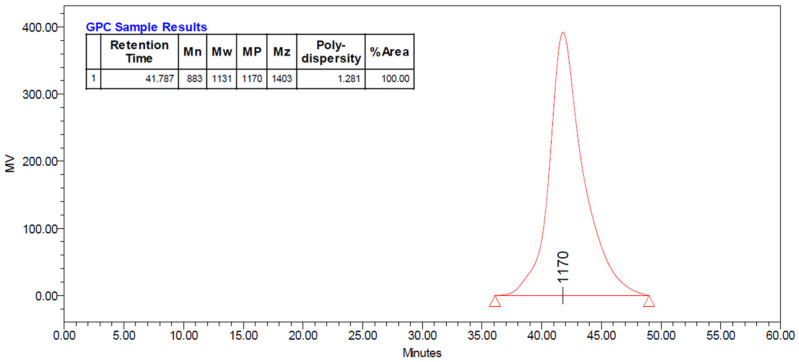
GPC results of HOL9 product.

The molecular weight distribution of the product was determined by GPC and is shown in Figure 1. The weight average molecular weight (M_V) of the functionalized siloxane and the polymer dispersion index (PDI) indicated that the molecular weight distribution of the product was relatively uniform.

**Figure 2:**
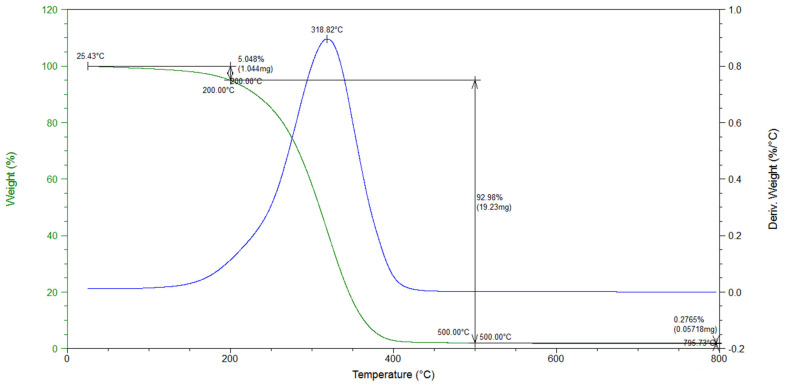
TGA results of HOL9 product.

The thermogravimetric analysis result of HOL9 is shown in Figure 2. The initial weight loss, attributable to the evaporation of solvents and other volatile components, signifies the onset of sample decomposition or evaporation (by 12.30%), is observed within the temperature range of 0 °C to 200 °C. The subsequent weight reduction signifies the thermal decomposition of the specimen by 88.00% up to approximately 500 °C. Within the temperature range of 500–800 °C, the weight loss is negligible (0.1446%).

The synthesized compound was then subjected to a differential scanning calorimetry (DSC) analysis. The DSC analysis of the HOL9 product is presented in Figure S2 of the Supplementary Materials. During the initial heating and cooling cycle, two distinct temperature-related signals were detected: an endothermic melting signal at 2.61 °C and an exothermic solidification signal at −18.66 °C. No additional signals indicating thermal processes in the sample were detected. It has been established that these thermal events are concomitant with phase transitions of the newly synthesized material. The significant discrepancy between the melting and crystallization temperatures suggests the occurrence of supercooling and potential kinetic constraints stemming from the amphiphilic character and molecular flexibility of the bifunctional siloxane derivative. The presence of hydrophilic polyether and hydrophobic alkyl substituents, as well as the permeability of the siloxane backbone, contribute to the compound’s complex thermal properties.

In summary, the TGA and DSC results confirm the presence of well-defined phase transitions that can be attributed to the molecular architecture of the synthesized material. Integrating spectroscopic (NMR), thermal (TGA and DSC), and microstructural (microscopic imaging) data facilitates a comprehensive evaluation of the properties of the synthesized composite and its effect on the microstructure and resistance to micro-organisms of the cement composite.

### 2.2. Results of Biological Tests for Cement Composite with Surface-Applied Compound

**Figure 3:**
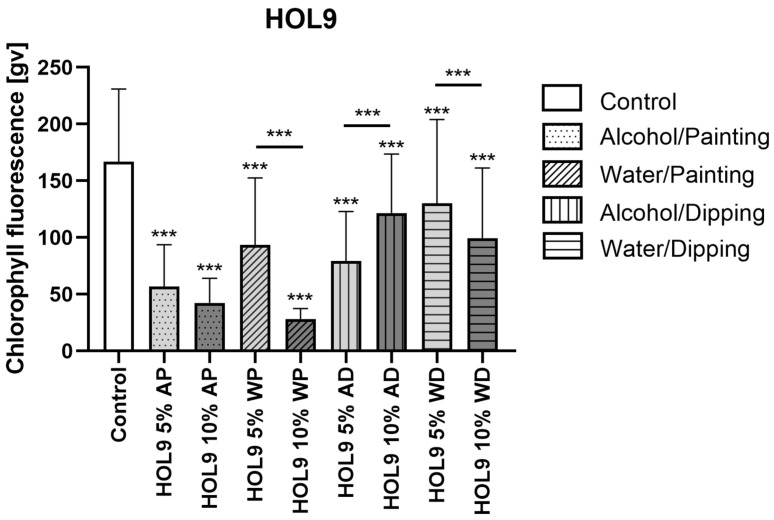
Changes in ^chl^FI in microalgal cells growing for 21 days on cement composite coated by painting or dipping, in two concentrations of HOL9 compound, i.e., 5% and 10% in alcohol or water solutions. *** *p* < 0.001.

In the initial phase, a series of biological tests was conducted on cement specimens with a *w*/*c* ratio of 0.50. The evaluation of chlorophyll fluorescence (^chl^FI) revealed a substantial (*p* < 0.001) decline in the photosynthetic activity of algal cells in comparison to the control cement composite. The observed reduction was found to be independent of the concentration of HOL9 (5% vs. 10%), the coating method (painting vs. dipping), and the type of solvent utilized (alcohol vs. water). However, the most significant decrease in algal growth was observed when the HOL9 compound was applied as a paint solution composed of 10% water (HOL9 10% WP). The maximum ^chl^FI value exhibited a 97.6% decrease, while the mean value decreased by 83.1% (see Figure 3 and Table 1). Furthermore, a substantial decline in chlorophyll activity was observed in response to the application of the 10% HOL9 alcohol solution by painting (see Figure 3 and Table 1).

**Figure 4:**
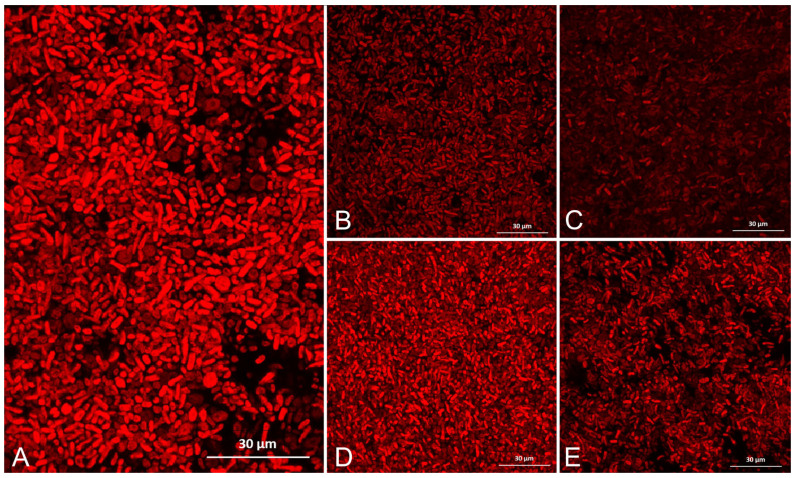
Changes in ^chl^FI in microalgal cells growing for 21 days on cement composite between control (**A**) and samples covered by the tested compound HOL9 in 5% concentration. (**B**,**C**) HOL9 in alcohol solution by dipping (**B**) and painting (**C**). (**D**,**E**) HOL9 in water solution by dipping (**D**) and painting (**E**).

**Figure 5:**
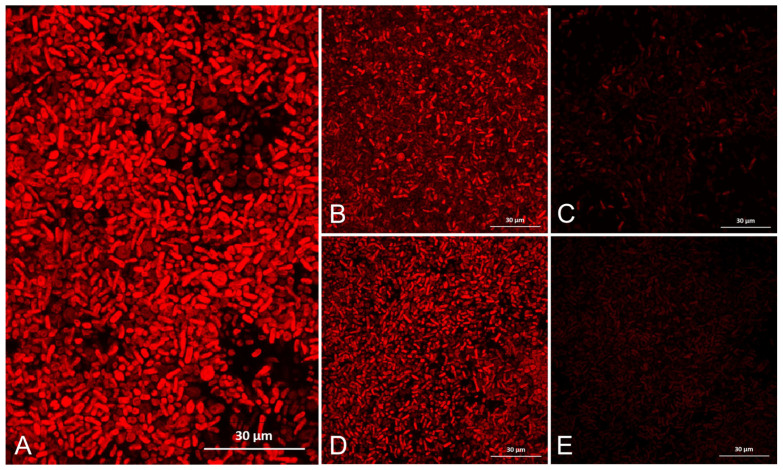
Changes in ^chl^FI in microalgal cells growing for 21 days on cement composite between control (**A**) and samples covered by the tested compound HOL9 in 10% concentration. (**B**,**C**) HOL9 in alcohol solution by dipping (**B**) and painting (**C**). (**D**,**E**) HOL9 in water solution by dipping (**D**) and painting (**E**).

The obtained results were confirmed by the microphotographs taken with confocal microscopes, indicating a decrease in ^chl^FI following the application of the HOL9 compound (see Figure 4 and Figure 5). The images reveal a significant reduction in fluorescence intensity when a 10% HOL9 solution is applied through painting, with water solutions showing a greater impact compared to alcohol solutions (Figure 3).

### 2.3. Surface Properties

**Figure 6:**
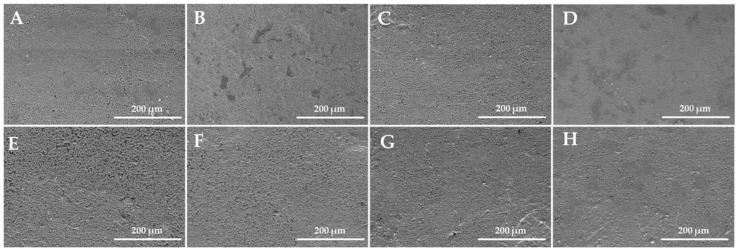
SEM images of cement composites samples with HOL9 modification: from 5% alcohol solution deposited by painting (**A**) and dipping (**C**), and from 5% water solution deposited by painting (**B**) and dipping (**D**), and from 10% alcohol solution deposited by painting (**E**) and dipping (**G**), and from 10% water solution deposited by painting (**F**) and dipping (**H**). Scale bars are 200 µm.

The cement composites were modified using a substance known as HOL9, which was deposited from a water or alcohol solution via two methods: dipping or painting. An analysis of the effect of solution type on the low-magnification morphology of cement composites indicates that water-based solutions invariably yield a flat sample surface composed of grains measuring a few micrometers in diameter and dense islands measuring tens of micrometers in diameter, irrespective of concentration (see Figure 6B,D,F,H). In contrast, alcohol solutions yield a surface that is significantly degraded, characterized by substantial grains measuring tens of micrometers in diameter and marked by high surface roughness (see Figure 6A,C,E,G). This effect is most noticeable on painted surfaces (see Figure 6B,F). The morphology at a magnification of 500 µm is presented in Figure S3 of the Supplementary Materials.

**Figure 7:**
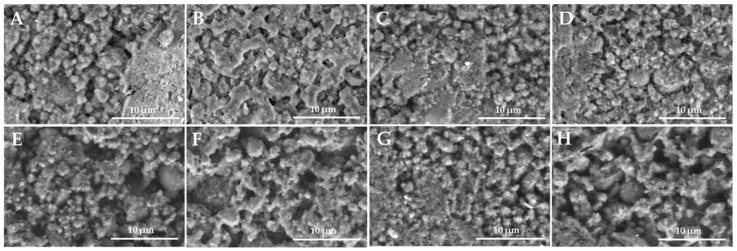
SEM images of cement composite samples with HOL9 modification: from 5% alcohol solution deposited by painting (**A**) and dipping (**C**), and from 5% water solution deposited by painting (**B**) and dipping (**D**), and from 10% alcohol solution deposited by painting (**E**) and dipping (**G**), and from 10% water solution deposited by painting (**F**) and dipping (**H**). Scale bars are 10 µm.

A comparison of microscale deposition methods demonstrates more precisely the effect of modifier solution concentration and the solvent used on the substrate structure (Figure 7). Water solutions have been observed to remove dendrite-like nanostructures and round individual grains, resulting in a smoother surface (Figure 7B,D,F,H). This effect is more pronounced with higher concentrations of HOL9 (see Figure 7F,H), particularly when the coating is applied by painting (see Figure 7F). A comparable outcome was attained through the utilization of a 10% solution concentration in water coatings by immersion (Figure 7H). Consequently, the SEM images presented herein reflect the results of biological studies that demonstrated the superior effectiveness of higher-concentration water solutions (Figure 3).

**Figure 8:**
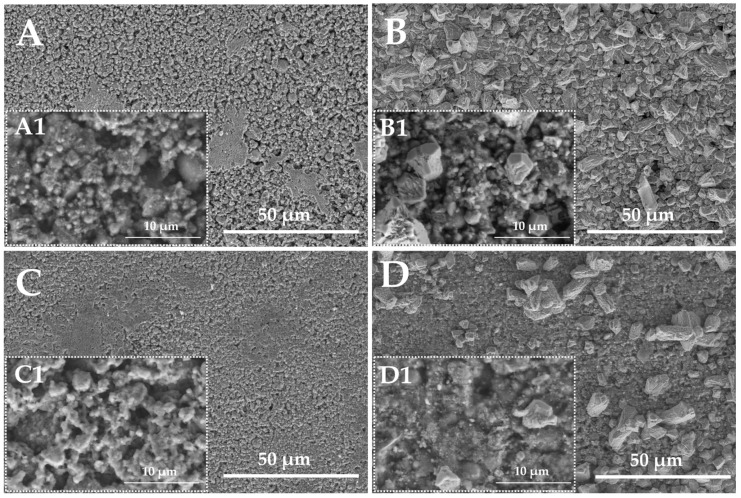
The effect of ageing on substrates modified by painting with either a 10% alcohol solution (**A**,**B**) or an water solution (**C**,**D**) is shown. The scale bar is 50 µm for (**A**,**B**), and 10 µm for (**A1**–**D1**).

The aging experiment, which entailed immersing the concrete samples in an alkaline solution, resulted in substantial alterations to their morphology (Figure 8). The predominant morphological aging effect is the formation of elongated structures measuring several dozen micrometers, as well as smaller, short grains with diameters of several micrometers (see Figure 8B,D). In the case of HOL9 samples modified with alcohol-based solutions, large, elongated structures cover the entire surface (Figure 8B). In the case of water solutions, the surface after aging consists of fewer, less developed, large, elongated structures (Figure 8D). This finding suggests that the durability of the coating applied by painting using a water solution may be enhanced (Figure 8D).

### 2.4. Physical and Mechanical Properties of Coated Cement Composite Samples

The next stage of the research involved carrying out physical and mechanical tests. The samples selected for testing were based on the results of the biological and morphological tests obtained. The correlations resulting from the protective coating application method were investigated further, and the results are summarised in Table 2.

The density of the samples that were coated with the HOL9 compound via the dipping method (D) and the painting method (P) was found to be comparable to that of the reference samples. The samples that were coated with the compound exhibited no alteration in water absorption, and the method of compound application also demonstrated no effect on water absorption. The compressive strength of the coated samples was found to be lower than that of the uncoated samples. However, it is noteworthy that all composites tested achieved a minimum compressive strength of 73.0 MPa when using 42.5 grade cement. The outcomes of the freeze–thaw cycling resistance test, which involved 150 cycles, substantiate the absence of an impact of the HOL9 compound application and the method of application on the strength of the reference and tested specimens. It was observed that the decrease in strength after undergoing cycles of freezing and thawing was analogous to the decrease in strength of samples that were not coated—control (a decrease of 42.8%)—and amounted to 50.7% for HOL9-D samples and 43.8% for HOL9-P samples, respectively.

## 3. Discussion

This publication details the synthesis and application of the functionalized organosilicon compound HOL9 as a surface modifier. A polyether substituent was successfully attached to a siloxane molecule via a controlled hydrosilylation reaction. This method yielded a straightforward, one-step procedure for synthesizing a compound that exhibited amphiphilic characteristics. The alkyl groups present in the siloxane are hydrophobic but chemically unreactive. Consequently, a reactive hydrophilic substituent was incorporated. The polyether substituent’s propensity for hydrolysis and subsequent condensation is attributable to the presence of hydroxyl groups, thereby facilitating the coating’s adhesion to the modified substrate. The functionalized compound HOL9 has the potential to serve as a substitute for alkylsilanes and polysiloxanes, which are frequently utilized in the literature for impregnating building materials [35,36,37].

Hydrophobic substances are widely recognized for their ability to resist fouling. These agents have been shown to reduce the adhesion strength between cells and surfaces, thereby facilitating cell-release characteristics [38,39]. However, it should be noted that hydrophobic surfaces are incapable of impeding the attachment of fouling organisms. In fact, certain microalgae have been observed to adhere more effectively to hydrophobic surfaces by secreting extracellular polymeric substances [40]. In contrast, hydrophilic materials have been shown to impede the initial colonization of microorganisms by creating a hydration layer [38]. The compound HOL9 contains both hydrophobic (methyl group) and hydrophilic (polyether group) elements in its structure, functioning more like an amphiphilic coating with dual properties. The efficacy of analogous compounds in managing algal fouling has been demonstrated by Wang et al. [41], who found that an amphiphilic compound significantly reduced the marine green alga spores on the surface of the material. In the context of the aerial environment, the primary factor determining a cell’s survival capabilities is the availability of water, specifically in the form of water vapor in the atmosphere. Aerial microalgae are poikilohydric organisms, meaning they are unable to regulate their water content independently. Limited access to water elevates the risk of cellular desiccation, leading to metabolic disruptions. Furthermore, it has been demonstrated that environmental and atmospheric conditions play a role in the process of desiccation. In addition, there is evidence indicating that hydrophilic surface coatings may contribute to the dehydration of algal cells [20].

The concentration of the HOL9 compound, as well as its method of application, was found to be the most significant factor influencing the reduction in chlorophyll content on the substrate under study. The most effective treatment evaluated by decreased fluorescence was a 10% water solution of HOL9, which was applied by painting the substrate. In a separate study involving another compound, HOL7, it was demonstrated that the alcohol-based solution applied by dipping was more effective [20]. However, in the case of HOL7, a glass substrate was used, and it could not absorb the compound deeply. In such instances, the liberation of the active compound occurs rapidly, akin to the behavior of painted cement composite [20]. This finding indicates that the optimal outcomes for silicon polyether compounds may be attained due to the compound’s direct action, rather than the prolonged effect of the compound’s release from a soaked substrate.

As is the case with other siloxanes, HOL9 bonds to the cement substrate in several stages. Initially, the siloxane undergoes a hydrolysis reaction, resulting in the formation of unstable silanolane when it comes into contact with water, either on the surface of the composite or in the ambient atmosphere. Subsequently, the substance undergoes a process of dehydration and condensation, resulting in the formation of polysiloxane during the drying phase. Consequently, the compound is bonded to the substrate through covalent bonding [28]. The catalytic effect is achieved due to the alkaline nature of the cement matrix [42]. The application of HOL9, which contains both hydrophilic and hydrophobic groups, does not significantly affect the properties of the cement composite. The material’s density and water absorption remain constant, although there is a slight decrease in compressive strength. Minor alterations in compressive strength are observed following the implementation of HOL9 after a 28-day period, which corresponds to the point at which the material is assumed to have attained its intended strength [43]. However, the observed variations in compressive strength may be attributed to the sealing of the surface and the subsequent limitation of the hydration process following the application of HOL9 and prior to the execution of the strength test. However, it is noteworthy that all samples exhibited strengths exceeding 73 MPa. Moreover, given the potency of the reference samples from the cyclic freeze–thaw testing, no discrepancy in compressive strength was detected in samples that were not subjected to cyclic freezing, contingent on the application of HOL9. It is noteworthy that the utilization of siloxane HOL9, given its structural characteristics and dual nature (hydrophobic/hydrophilic), leads to divergent trends in the mechanical and frost resistance properties of the composite compared to the application of prevalent silanes. The majority of silanes exhibit a pronounced hydrophobic effect, thereby enhancing frost resistance [44,45]. A comprehensive review of the extant literature on the subject indicates that the method of applying the coating, the concentration of the modifier, and the type of solvent used have a significant impact on the properties of the surface obtained. Scanning electron microscopy (SEM) images clearly demonstrate that the substrate structure changes depending on the modification method, which in turn indirectly affects the mechanical properties, frost resistance, and effectiveness of surface protection against phototropic colonization of cement composite. However, the HOL9 compound does not induce this effect. Furthermore, the implementation of the compound, particularly through the method of dipping, has the potential to induce a degradation in the resistance to repetitive freezing and thawing cycles.

## 4. Materials and Methods

### 4.1. Materials

#### 4.1.1. Functionalized Silicone HOL9

All commercially available chemicals were used without further purification. 1,1,1,3,5,5,5-heptamethyltrisiloxane was purchased from Gelest (Arlington, VA, USA). Allyl polyether (BIKANOL 9) was purchased from ICSO Chemical Production, Kędzierzyn-Koźle, Poland. Karstedt’s catalyst, which is a commercially available hydrosilylation catalyst, was purchased from Sigma-Aldrich (Darmstadt, Germany).

#### 4.1.2. Composition and Preparation of Cement Composite

The samples were prepared using CEM I 42.5 R (Górażdże S.A., Dąbrowa Górnicza, Poland), a hydraulic binder obtained by co-grinding Portland clinker (the main component) with sulfate-based materials that regulate the setting time. This cement conforms to the specifications delineated in EN 197-1 [46]. The quartz sand utilized in the cement composites functions as a fine aggregate, with grain sizes reaching up to 2 mm in diameter. This fine aggregate meets the requirements specified in EN 196-1 [47]. The cement composite was prepared using distilled water. This distilled water is in accordance with the standards specified in EN 1008 [48].

All samples were prepared in accordance with PN-EN 196-1 [47], employing a standard mix ratio of 1:3 (cement-to-sand) with a *w*/*c* ratio of 0.50. The materials and equipment were subjected to a temperature adjustment process to ensure they reached the desired laboratory temperature. All components were meticulously weighed with a precision of ±1 g. The mixture was initiated with water, followed by cement (administered at a low speed for 30 s), and subsequently sand (incorporated over a duration of 30 s). Following a 30 s period of high-speed mixing, the process was paused for a duration of one minute and 30 s. Thereafter, the process was resumed at a high speed for a period of 60 s. The samples were subsequently placed in 40 × 40 × 160 mm molds, with the composite being distributed across two compacted layers, with each layer comprising 60 shakes. Subsequent to leveling, the samples are maintained within the mold for a period of 24 h. Thereafter, following the process of demolding, the samples are placed in water for a duration of 27 days. The samples were subjected to coating after a 28-day period of curing, followed by testing.

### 4.2. Methods

#### 4.2.1. Synthesis of HOL9

The synthesis of the HOL9 compound was carried out based on a typical hydrosilylation reaction. The reaction was conducted in an open system, in the presence of toluene as a solvent and using a Karstedt platinum catalyst. A siloxane containing a polyether group was synthesised in a hydrosilylation reaction involving 1,1,1,3,5,5,5-heptamethyltrisiloxane and an allyl polyether containing nine ethoxy groups and a terminal hydroxy group. The following reagents were meticulously transferred into a three-necked round-bottom flask, which was then equipped with a reflux condenser, thermometer, and magnetic stirrer: 16.46 g (74 mmol) of 1,1,1,3,5,5,5-heptamethyltrisiloxane; 33.60 g (74 mmol) of allyl polyether; 21.5 µL (3 × 10^−5^ mol Pt/1 mol Si–H) of Karstedt catalyst; and 50 g of toluene. The contents of the flask were stirred continuously as the mixture was introduced dropwise. The mixture was subsequently subjected to a process temperature of 110 °C. The reaction was monitored using infrared spectroscopy to observe the disappearance of the 904 cm^−1^ band, which is assigned to the Si-H bond in the substrate. Subsequent to the completion of the process, the post-reaction mixture was cooled, and the products were isolated by distilling off the solvent and excess olefin under reduced pressure. The pure product was obtained in high yield (98%). Subsequently, the products were subjected to spectroscopic analysis to verify the assumed structure.

#### 4.2.2. HOL9 Application

Solutions comprising functionalized HOL9 siloxane, along with water or isopropanol as a solvent, were meticulously prepared at concentrations of 5% and 10%. In order to obtain a homogeneous mixture, it was necessary to mix the solution components at room temperature for a period of 30 min. The impregnants were prepared by subjecting the cement composite blocks (40 × 40 × 160 mm) to an immersion process for 30 min or by applying two coats of a wet-on-wet coating method. Subsequent to this interval, the blocks were subjected to air-drying until a constant mass was achieved. Thereafter, the blocks were subjected to additional testing.

#### 4.2.3. Physicochemical Characterization of Compound HOL9 and the Coatings

Magnetic nuclear resonance spectra (^1^H NMR, ^13^C NMR, and ^29^Si NMR) were obtained on a Bruker Ascend 400 at room temperature using CDCl_3_ as the solvent.

The FT-IR spectra were obtained using a Nicolet iS20 mid-infrared FT-IR spectrometer (ThermoFisher Scientific, Waltham, MA, USA) equipped with a diamond ATR attachment. Spectra were collected in the range of 500–4000 cm^−1^, with a resolution of 2 cm^−1^, and 32 scans of the background and sample were recorded. The progression of the reaction was quantified by observing the rate of change in the area of the band with a maximum at 904 cm^−1^, which is assigned to the stretching vibrations of Si–H.

Thermogravimetric analysis (TGA) was conducted using a Q50 apparatus (TA Instruments, New Castle, PA, USA) under a nitrogen flow of 60 mL/min. The temperature was increased from room temperature to 800 °C at a heating rate of 10 °C/min.

Differential scanning calorimetry (DSC) measurements were performed on a DSC1 instrument (Mettler Toledo, Parramatta, Australia). Analyses were conducted in an argon atmosphere at a constant flow rate of 20 milliliters per minute. The samples were meticulously weighed and subsequently placed within aluminum crucibles. The results were then subjected to analysis using the STAR^®^ software (https://www.mt.com/pl/pl/home/library/product-brochures/lab-analytical-instruments/STARe_software_Brochure.html, accessed on 2 February 2026) provided by Mettler Toledo.

The morphological analysis of concrete modified by molecular agents was performed using a Quanta FEG 250 (FEI) scanning electron microscope (SEM) with a beam energy of 10 keV. The SEM was operated in low vacuum mode at a pressure of 70 Pa.

#### 4.2.4. Absorption Test

The test, as delineated in P-B-04500 [49], quantifies the water absorption of a specimen submerged under standard atmospheric pressure. The experiment is conducted on three specimens measuring 40 × 40 × 160 mm. Following saturation, the samples undergo desiccation to a constant weight, thereby ensuring that the weight difference between 24 h intervals does not exceed 0.2%.

#### 4.2.5. Density

The test comprises the weighing and measuring of three 40 × 40 × 160 mm bales to ascertain their volume and density in accordance with PN-EN 1015-10 [50].

#### 4.2.6. Compressive Strength Test

The compressive strength test was conducted in accordance with the provisions of PN-EN 196-1 [47] by positioning half of the beam between square pads. The 40 × 40 mm pads were composed of hardened steel. The load was subsequently applied to the central part of the beam at a constant rate of 2.4 ± 0.2 kN/s until the sample failed.

#### 4.2.7. Freez-Thaw Test

The test was conducted in accordance with the provisions of PN-B-06265 [51]. The test involved exposing six samples, which were pre-weighed, water-saturated, and matured, to a series of 150 cycles of air-freezing and water-thawing. Freezing has been observed to occur at −18 ± 2 °C for a minimum of 4 h, while thawing has been recorded at 18 ± 2 °C for a duration of 2 to 4 h. Concurrently, six control specimens are maintained in water or at >90% relative humidity at 18 ± 2 °C. Subsequent to the completion of the predetermined number of cycles or upon the observation of evident damage, the specimens are weighed and subjected to testing for compressive strength, inclusive of the control samples.

#### 4.2.8. Biological Testing

The microalgal colonization assay was performed using a mixed culture of four green microalgal strains that are known to thrive on mineral substrates such as stone, concrete, cement, and plaster, as well as in terrestrial habitats, and they commonly occur on mineral building materials [5]. The following species have been identified: *Chlorodium saccharophilum* PNK010, *Pseudostichococcus monallantoides* PNK037, *Trebouxia aggregate* PNK080, and *Klebsormidium flaccidum* PNK013/2. Given the co-occurrence of these taxa in terrestrial biofilms, a mixed microalgal culture consisting of equal proportions of the strains was utilized for the study. Cement composite samples, both untreated (control) and treated with the HOL9 compound, were inoculated with 0.5 mL of the algal suspension and incubated under controlled laboratory conditions for 21 days. The incubation was conducted under artificial light with a day/night cycle and constant temperature and humidity, as previously described [20].

Following the incubation period, the biofilm was meticulously removed from the surface using a soft, sterile brush. The physiological state of the microalgal cells was assessed by measuring chlorophyll fluorescence intensity (^chl^FI), which serves as an indicator of photosynthetic activity. Fluorescence measurements were performed using a Leica TCS SP8 confocal laser scanning microscope (Leica Microsystems, Wetzlar, Germany) with excitation at 488 nm and emission detection in the range of 620–670 nm [52]. For each sample, 225 measurements were recorded and subsequently analyzed using LAS-AF 3.3.0.10134 software. The distribution of the data was then subjected to a test of normality using the Shapiro–Wilk test. Given the absence of a normal distribution in the data, non-parametric tests were employed. The statistical analysis of the differences between the two groups was conducted using the Mann–Whitney U test, while multiple comparisons were performed using the Kruskal–Wallis test with Bonferroni post hoc correction. Statistical analyses were conducted using GraphPad 8.0.1 Prism software.

#### 4.2.9. Alkali Resistance Testing

Tests for alkali resistance were conducted 14 days after HOL9 impregnation. The samples, which had been meticulously weighed, were then immersed in demineralized water. Subsequent to a 24 h period, the samples were retrieved, desiccated with a paper towel to eliminate residual water, and reweighed. Subsequently, the samples were transferred to a secondary container containing a potassium hydroxide solution with a concentration of 5.6 g/L. This solution was maintained for a duration of 21 days. In both cases, the samples were completely submerged in the liquid. Following a three-week period, the samples were extracted from the alkaline solution, weighed, and dried under a fume hood. Subsequent to attaining a constant weight, the initial cycle was reiterated with immersion in demineralized water.

## 5. Conclusions

Subsequent to the hydrosilylation reaction, functionalised siloxane HOL9 was obtained, which was then used for the impregnation of cement composites. Combining the spectroscopic (NMR) and thermal (TGA and DSC) data of the synthesised compound with the results of the mechanical and microstructural (SEM) microscopic imaging enabled a comprehensive assessment of the properties of the HOL9 compound and its impact on the microstructure and resistance of the cement composite to microorganisms and frost. A series of tests was conducted to ascertain the impact of the concentration of the active agent, the type of solvent, and the method of coating application on the mechanical properties, frost resistance, and effectiveness of protection against algae. The most significant decrease in algal photosynthetic activity was observed for the 10% aqueous HOL9 solution that was applied by painting. Under these conditions, the maximum chlorophyll fluorescence intensity (^chl^FI) value decreased by 97.6%, while the mean value decreased by 83.1% compared to the control. It was also demonstrated that the type of solvent used has a significant impact on the protective properties of the coating. Surface morphology changes significantly depending on the solvent used. Coatings based on alcohol appear to degrade significantly more. Elongated structures measuring several dozen micrometres in diameter cover virtually the entire sample surface. As a result of the above tests, the best results were obtained using a 10% aqueous solution of HOL9, therefore these samples were subjected to further testing. The density of samples coated with HOL9 using the immersion (D) and painting (P) methods was comparable to that of the reference samples (2.166 g/cm^3^), at 2.117 g/cm^3^ (D) and 2.136 g/cm^3^ (P), respectively. Samples coated with the compound showed no significant changes in water absorption, and the application method also had no effect, with absorption remaining at approximately 7%. Samples coated with HOL9 by either painting or immersion exhibited slightly lower strength than the reference sample. Nevertheless, regardless of the coating method used, all samples achieved an average minimum compressive strength of 73.0 MPa. The results of the freeze–thaw resistance test, comprising 150 cycles, confirmed that the application of HOL9 and the application method had no effect on the strength of the reference and test samples. Decreases in strength after freeze–thaw cycling were observed to be analogous to those of the uncoated control samples (a 42.8% decrease), amounting to 50.7% for the HOL9-D samples and 43.8% for the HOL9-P samples, respectively. These results suggest that, regardless of the impregnation technique used, the substrate retains its original strength and frost resistance compared to unmodified samples.

The permeation of vapours and gases by the coating is due to its siloxane structure and the absence of substituents at each atom. This property enables unobstructed air circulation. This property substantially enhances the ability to protect building materials from harmful external factors. The presence of polyether groups with terminal hydroxyl groups that readily hydrolyse and then condense enables the coating to adhere to the substrate. The protective effect is due to the structure of the siloxane compound and the carefully selected functional groups (hydrophobic and hydrophilic). The physiological inertness and non-toxicity of siloxanes, coupled with the elimination of the need for harmful biocides to protect the substrate from algae damage, make the proposed solution environmentally friendly.

Results demonstrate that the amphiphilic siloxane coating effectively suppresses the photosynthetic activity of algae, protecting cement composites against phototrophic colonisation while preserving key mechanical and durability properties. This highlights the coating’s potential as an environmentally friendly surface treatment. This publication marks the beginning of a series of studies that will investigate these technologies further.

## Figures and Tables

**Table 1:** General statistics of ^chl^FI measurements (no. = 225) in microalgal cells growing for 21 days on cement composite coated by painting or dipping in two concentrations of HOL9 compound, i.e., 5% and 10% in alcohol or water solutions *w*/*c* ratio = 0.50.

	Control	Alcohol/Painting		Water/Painting		Alcohol/Dipping		Water/Dipping	
		HOL9 5%	HOL9 10%	HOL9 5%	HOL9 10%	HOL9 5%	HOL9 10%	HOL9 5%	HOL9 10%
Maximum	255.0	145.5	96.2	218.1	57.1	138.5	250.7	253.3	254.7
Minimum	26.4	4.9	7.2	9.0	6.1	4.4	3.5	7.7	7.7
Mean	166.9	56.8	42.3	93.5	28.2	79.3	121.6	130.0	99.4
Median	169.9	53.0	37.8	83.0	28.0	99.0	129.3	131.5	83.9
Q1	126.7	24.5	24.0	38.7	21.1	73.8	114.3	75.6	55.3
Q3	222.6	82.9	56.8	148.8	34.3	108.2	143.5	190.9	147.9
Variance	4096.3	1364.5	471.6	3504.1	84.0	1911.3	2704.9	5477.0	3825.9
SD	63.9	36.9	21.7	59.1	9.1	43.6	51.9	73.8	61.7
*p*-value	-	<0.001	<0.001	<0.001	<0.001	<0.001	<0.001	<0.001	<0.001

**Table 2:** Physical and mechanical properties of cement composite samples with *w*/*c* = 0.5, where Control—without coating, D—coating by dipping, P—coating by painting.

	Density [g/cm^3^]		Water Absorption [%]		Compressive Strength [MPa]		Freezing-Thawing Resistance				Percentage Decrease in Strength
							Reference [MPa]		Tested [MPa]		
	Mean	SD	Mean	SD	Mean	SD	Mean	SD	Mean	SD	[%]
Control	2.166	0.008	7.094	0.079	84.2	1.8	64.8	1.1	37.1	2.0	42.8
HOL9-D	2.117	0.002	6.968	0.063	78.2	2.5	67.6	1.9	33.2	2.6	50.7
HOL9-P	2.136	0.009	7.190	0.174	73.0	3.7	68.6	1.4	38.6	3.1	43.8

## Data Availability

The original contributions presented in this study are included in the article/Supplementary Material. Further inquiries can be directed to the corresponding author.

## Supplementary Materials

The following supporting information can be downloaded at: https://www.mdpi.com/article/10.3390/ijms27031586/s1.
