# Supplementary material for: Functionalized Siloxane Coating as Protection of the Surface of Cement Composites Against Phototropic Colonization
Source: Int J Mol Sci. 2026 Feb 5;27(3):1586. doi: 10.3390/ijms27031586 (PMC12898598; doi:10.3390/ijms27031586)
Supplement: Supplementary file 1 [file ijms-27-01586-s001.zip › Figure S1.pdf]

Functionalized siloxane coating as protection of the surface of cement composites against phototropic colonization.

Joanna Karasiewicz[1*], Marta Thomas[2], Paulina Nowicka-Krawczyk[3], Rafał M. Olszyński[3], Piotr K. Zakrzewski[4], Agnieszka Ślosarczyk[2]

[1] Faculty of Chemistry, Adam Mickiewicz University in Poznań, Uniwersytetu Poznańskiego 8, 61-614 Poznan, Poland; (JK) joanna.karasiewicz@amu.edu.pl

[2] Faculty of Civil and Transport Engineering, Institute of Building Engineering, Poznan University of Technology, 60-965 Poznan, Poland; (MT) marta.thomas@put.poznan.pl,(AŚ) agnieszka.slosarczyk@put.poznan.pl

[3] Department of Algology and Mycology, Faculty of Biology and Environmental Protection, University of Lodz, Banacha 12/16. 90-237 Lodz, Poland; (PNK) paulina.nowicka@biol.uni.ludz.pl,(RMO) rafal.olszynski@biol.uni.ludz.pl

[4] Department of Molecular Neurochemistry, Medical University of Lodz, 92-215 Lodz, Poland

* Correspondence: joanna_karasiewicz@amu.edu.pl; Tel.: +48 618291733

The substrate and product spectra for the example compound HOL9 shown in Figure S1. A band at ύ= 3500 cm^{-1}, which can be attributed to stretching vibrations of the hydroxyl group. The presence of this band in the product spectrum and the disappearance of the bands at ύ= 2100 and 903 cm^{-1}, due to stretching vibrations of the Si-H group (present in the spectrum of the parent compound), testify to the formation of the hydrosilylation product and not to the occurrence of a condensation reaction between the Si-H and OH groups. The structure of the product was also confirmed by the presence of bands attributed to symmetric and asymmetric stretching vibrations characteristic of the C-H bonds of methyl and methylenic groups in the region ύ = 2700 - 3000 cm^{-1}, as well as bands attributed to C-H bonds of methyl and methylenic groups in the region ύ = 2700 - 3000 cm^{-1}, as well as bands attributed to stretching vibrations of C-O-C bonds present in the polyether chains and asymmetric stretching vibrations of Si-O-Si groups in the region ύ= 1000 - 1200 cm^{-1}.

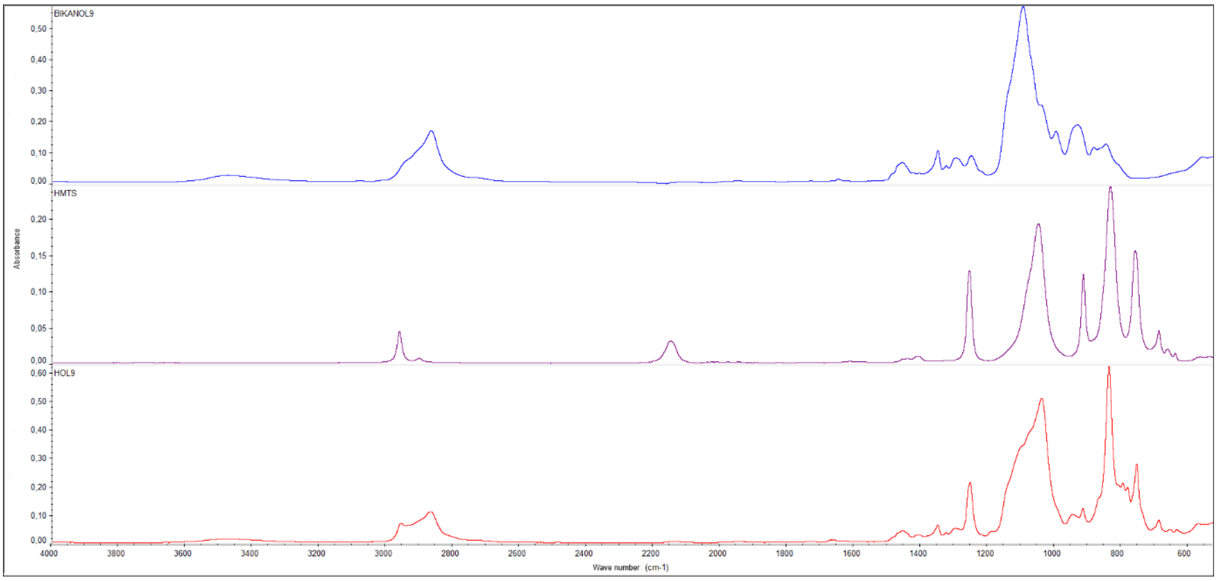

Figure S1. FT-IR spectra of the parent compounds and the HOL9.
