# Supplementary material for: Functionalized Siloxane Coating as Protection of the Surface of Cement Composites Against Phototropic Colonization
Source: Int J Mol Sci. 2026 Feb 5;27(3):1586. doi: 10.3390/ijms27031586 (PMC12898598; doi:10.3390/ijms27031586)
Supplement: Supplementary file 1 [file ijms-27-01586-s001.zip › Figures S2 and S3.pdf]

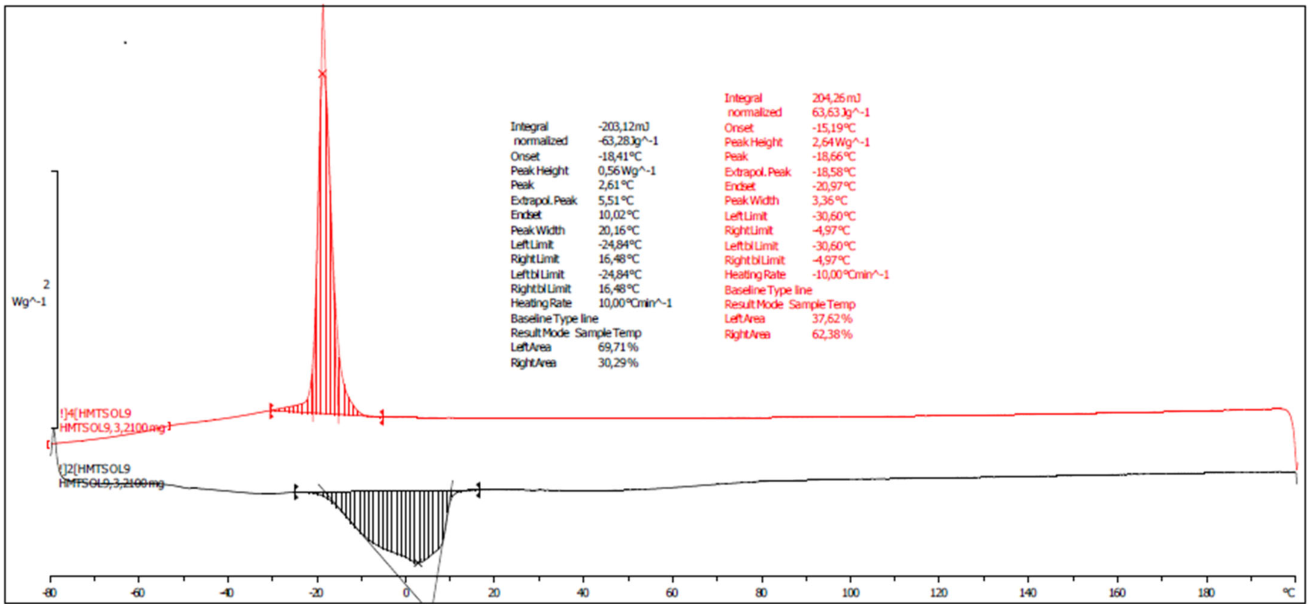

Figure S2. The DSC analysis of the HOL9 product.

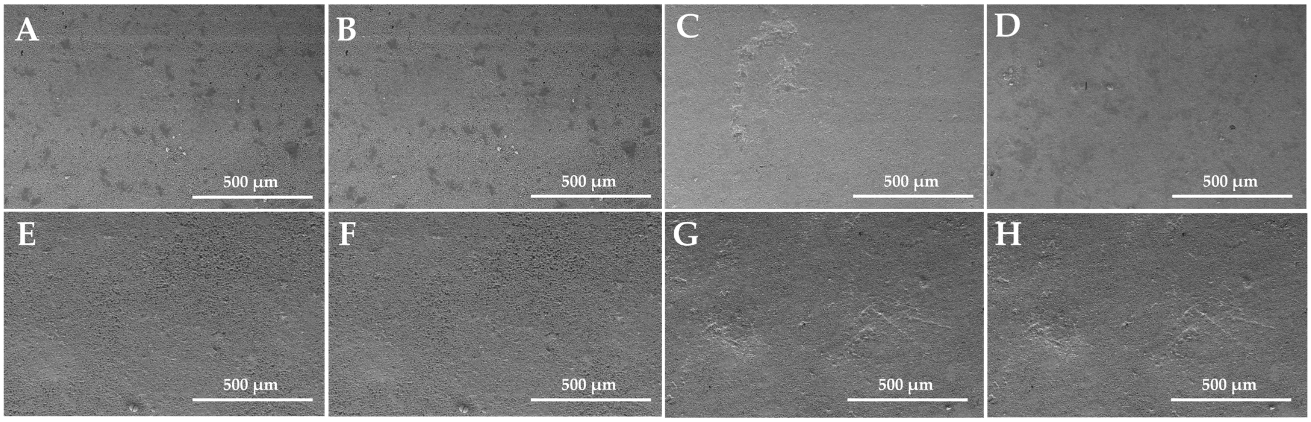

Figure S3. SEM images of cement composites samples with HOL9 modification: from 5% alcohol solution deposited by painting (**A**) and dipping (**C**), and from 5% water solution deposited by painting (**B**) and dipping (**D**), and from 10% alcohol solution deposited by painting (**E**) and dipping (**G**), and from 10% water solution deposited by painting (**F**) and dipping (**H**). Scale bars are 500 µm.
